# Preliminary exploration of the optimal timing of chemoimmunotherapy combined with radiotherapy for oligometastatic esophageal squamous cell carcinoma and analysis of prognostic factors: a multicenter retrospective study

**DOI:** 10.3389/fonc.2026.1734354

**Published:** 2026-05-18

**Authors:** Tian Xu, Chuanyu You, Xueping Yang, Xinyu Chen, Qilin Wang, Juncai Ye, Guangzeng Zhou, Jiahao Chen, Yan Gui

**Affiliations:** 1 Department of Oncology, Affiliated Hospital of North Sichuan Medical College, Nanchong, China; 2 Clinical College, North Sichuan Medical College, Nanchong, China

**Keywords:** esophageal squamous carcinoma, immunotherapy, oligometastasis, optimal timing, radiotherapy, systemic therapy

## Abstract

**Objectives:**

To explore the optimal timing of chemoimmunotherapy (CIT) combined with radiotherapy (RT) for oligometastatic esophageal squamous cell carcinoma (ESCC) and analyze prognostic factors.

**Methods:**

In this multicenter, retrospective study, we reviewed 142 patients with oligometastatic ESCC who received first-line RT and CIT between 2019 and 2024. Based on the timing of RT initiation relative to CIT, patients were categorized into three groups: RT-first (n=27), Concurrent (RT within the first 4 cycles of CIT, n=87), and Sequential (RT after 4 cycles of CIT, n=28). Inverse probability of treatment weighting (IPTW) and multivariable Cox regression were utilized to mitigate baseline confounding.

**Results:**

Following IPTW adjustment, baseline characteristics were well-balanced. The Concurrent group demonstrated a significantly superior overall survival (OS) compared to the RT-first and Sequential groups (median OS: 22.7 vs. 21.0 vs. 18.1 months, respectively; P = 0.018). Multivariable analysis confirmed that the concurrent strategy was an independent protective factor for OS (HR = 0.595, 95% CI: 0.354–0.999, P = 0.049). Progression-free survival (PFS) and anatomical patterns of first failure did not differ significantly among the three cohorts. Furthermore, the incidence of Grade ≥ 3 treatment-related adverse events was comparable across all groups (P > 0.05).

**Conclusions:**

In patients with oligometastatic ESCC, concurrent integration of RT within the early cycles of first-line chemoimmunotherapy is significantly associated with prolonged overall survival, without increasing severe toxicity, compared to upfront or delayed RT. These findings provide compelling real-world evidence to guide optimal treatment sequencing, warranting further validation in prospective trials.

## 1 Introduction

According to the 2022 National Cancer Report, Esophageal Cancer (EC) remains a major public health burden in China, ranking as the seventh most common malignancy and the fifth leading cause of cancer-related mortality in China (1). Due to its insidious onset, the majority of patients are diagnosed at a locally advanced or metastatic stage. Even with guideline-recommended first-line therapies, the median overall survival (mOS) remains poor, typically ranging from 12.4 to 17.2 months (2). Oligometastatic EC is generally considered an intermediate state between localized and widely metastatic disease (3), with reported 3-year and 5-year actuarial survival rates of 59.5% and 51.7%, respectively (4). The currently accepted definition of oligometastatic esophageal squamous cell carcinoma (ESCC) is: ≤5 metastatic sites distributed across ≤3 organs, or metastasis to a single regional lymph node (5–7).

In recent years, the standard of care for advanced ESCC has fundamentally shifted to immune checkpoint inhibitors (ICIs) combined with chemotherapy (chemoimmunotherapy, CIT). However, systemic therapy alone rarely achieves durable disease control in macroscopic lesions, often leading to acquired resistance and disease progression. A growing body of clinical evidence suggests that integrating local therapy—particularly radiotherapy (RT)—into the CIT backbone can significantly improve outcomes for oligometastatic ESCC (8–10). Radiotherapy not only achieves local cytoreduction but also acts as an immunological adjuvant. Radiation-induced tumor cell death releases tumor neoantigens and pro-inflammatory cytokines, modifying the tumor microenvironment and triggering an “*in situ* vaccine” effect that is associated with the potentiation of the systemic efficacy of ICIs. Consequently, European clinical practice guidelines for oligometastatic esophageal cancer now endorse the incorporation of RT with CIT (11).

Despite this theoretical synergy, an extensive review of the current literature reveals a critical knowledge gap regarding the optimal temporal sequencing of RT and CIT. The timing of RT integration—whether upfront, concurrently with early cycles of CIT, or sequentially as consolidation—remains highly controversial. Biologically, upfront or extensive RT may induce severe radiation-induced lymphopenia, depleting the circulating effector T-cells required for ICI efficacy (12, 13). Conversely, delaying RT too long may miss the optimal window for immune priming and be linked to an increased risk of systemic dissemination. While landmark studies in other thoracic malignancies (e.g., the PACIFIC trial) (14) have underscored the profound impact of chemoradiotherapy sequencing on survival, specific evidence in oligometastatic ESCC is sparse and contradictory. Several phase II trials and small retrospective series have explored either concurrent or sequential approaches independently, including studies such as PALACE-1, Duan et al., and Li et al. (5, 9, 15) yet direct comparative analyses evaluating the exact clinical timing of RT initiation within a real-world first-line CIT setting are virtually non-existent.

While systemic chemoimmunotherapy has become the standard first-line treatment for metastatic ESCC, the integration of local radiotherapy remains an area of active investigation. Several recent retrospective series have provided evidence for the benefit of adding RT to systemic regimens. For instance, Amin et al. (2024) demonstrated that the addition of RT to immunotherapy and chemotherapy significantly improved overall survival in patients with stage IV esophageal cancer, supporting the survival benefit of combined-modality treatment in advanced disease (16). Similarly, an analysis of the National Cancer Database by Tasoudis et al. (2024) demonstrated the feasibility and survival benefit of incorporating immunotherapy into multimodal chemoradiation strategies, further supporting the clinical value of combined-modality treatment (17). Furthermore, Wu et al. (2023) confirmed that CIT combined with RT offers superior outcomes compared to CIT alone in real-world metastatic ESCC cohorts (18). Collectively, these retrospective and real-world studies consistently support the clinical value of integrating radiotherapy into systemic immunotherapy-based strategies; however, they primarily focused on the presence or absence of radiotherapy rather than the optimal timing of its delivery. Importantly, none of these studies evaluated the comparative efficacy of different radiotherapy sequencing strategies within the same cohort, leaving the clinically relevant question of optimal RT timing largely unanswered.

However, despite the accumulating evidence supporting RT integration, the optimal temporal sequencing of radiotherapy—specifically whether RT should be delivered before, during, or after systemic chemoimmunotherapy—remains a major unresolved clinical question. Most previous studies evaluated concurrent or sequential approaches independently, without performing direct head-to-head comparisons among multiple timing strategies within the same clinical cohort.

Therefore, this multicenter real-world study was designed to directly compare survival outcomes and failure patterns among three distinct radiotherapy sequencing strategies: RT-first, concurrent RT, and sequential RT. By leveraging inverse probability of treatment weighting (IPTW) to mitigate baseline confounders, this study aims to evaluate the optimal sequence for integrating RT with CIT in oligometastatic ESCC, thereby providing clinically relevant evidence to inform the optimal integration of radiotherapy into first-line chemoimmunotherapy for oligometastatic ESCC.

## 2 Materials and methods

### 2.1 Study design and patient population

**Figure 1:**
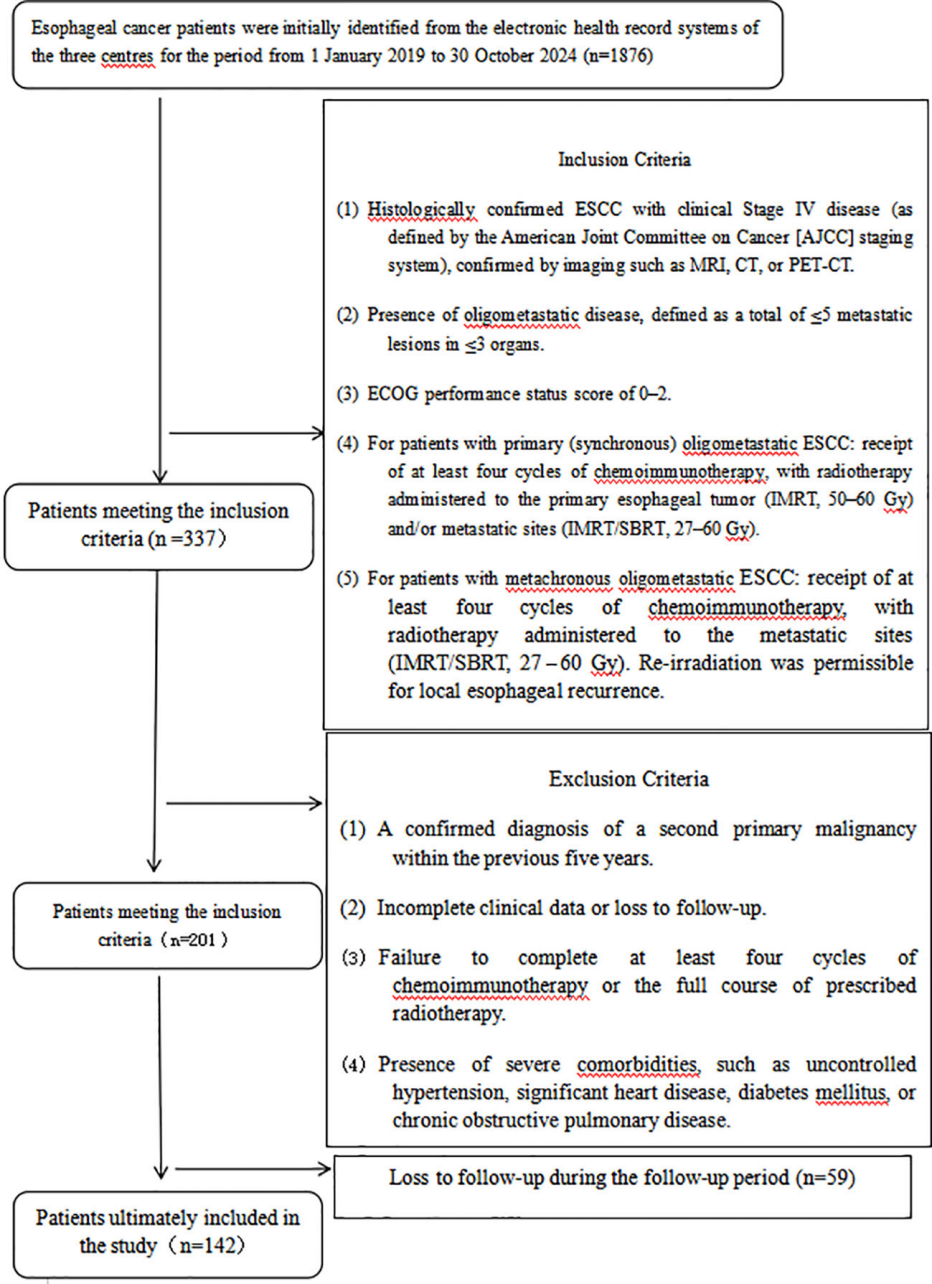
STROBE flowchart.

This was a multi-center, retrospective study. We included patients with oligometastatic esophageal squamous cell carcinoma (ESCC) who were treated between January 1, 2019, and October 30, 2024, at three tertiary hospitals in China: the Affiliated Hospital of North Sichuan Medical College, Nanchong Central Hospital, and Suining Central Hospital. The study cohort consisted of patients with any of the nine classes of oligometastatic disease as defined by the joint expert consensus of the European Society for Radiotherapy and Oncology (ESTRO) and the European Organization for Research and Treatment of Cancer (EORTC). A detailed flowchart of the patient selection process is provided in the STROBE diagram (**Figure 1**).

We initially screened the institutional databases to identify a preliminary cohort of patients diagnosed with ESCC. To establish the final study cohort, patients were strictly required to meet all of the following Inclusion Criteria (1): Histologically confirmed ESCC with clinical Stage IV disease (as defined by the American Joint Committee on Cancer [AJCC] staging system), confirmed by imaging such as MRI, CT, or PET-CT (2). Presence of oligometastatic disease, defined as a total of ≤5 metastatic lesions in ≤3 organs, or metastasis to a single regional lymph node (3). An Eastern Cooperative Oncology Group (ECOG) performance status score of 0–2 (4). For patients with primary (synchronous) oligometastatic ESCC: receipt of at least four cycles of chemoimmunotherapy, with radiotherapy administered to the primary esophageal tumor (IMRT, 50–60 Gy) and/or metastatic sites (IMRT/SBRT, 27–60 Gy) (5). For patients with metachronous oligometastatic ESCC: receipt of at least four cycles of chemoimmunotherapy, with radiotherapy administered to the metastatic sites (IMRT/SBRT, 27–60 Gy). Re-irradiation was permissible for local esophageal recurrence.

Subsequently, patients who met any of the following conditions were removed from the cohort based on the Exclusion Criteria (1): A confirmed diagnosis of a second primary malignancy within the previous five years (2). Incomplete clinical data or loss to follow-up (3). Failure to complete at least four cycles of chemoimmunotherapy or the full course of prescribed radiotherapy (4). Presence of severe comorbidities, such as uncontrolled hypertension, significant heart disease, diabetes mellitus, or chronic obstructive pulmonary disease.

Some patients with oligometastatic esophageal cancer present with significant symptoms from their metastatic lesions, for whom upfront radiotherapy may be administered for palliative relief. This study protocol was approved by the Institutional Ethics Review Committee (Approval No. 2025ER206-1). Due to the retrospective nature of the study, the requirement for individual patient informed consent was waived. All procedures were conducted in accordance with the ethical principles of the Declaration of Helsinki. Based on the timing and sequence of radiotherapy relative to chemoimmunotherapy, patients were categorized into three groups: the RT-first group (radiotherapy administered before the start of chemoimmunotherapy, n = 27), the Concurrent group (radiotherapy administered within the first four cycles of chemoimmunotherapy, n = 87), and the Sequential group (radiotherapy administered after the completion of four cycles of chemoimmunotherapy, n = 28).

### 2.2 Treatment protocols

#### 2.2.1 Systemic chemoimmunotherapy

Rather than a generalized approach, all 142 patients in this retrospective cohort received specific first-line systemic chemoimmunotherapy based on institutional standards and individual patient profiles. The immunotherapy backbone predominantly consisted of PD-1 inhibitors, specifically camrelizumab (n = 96), tislelizumab (n = 40), or sintilimab (n = 6), administered intravenously every 3 weeks. Concurrent chemotherapy regimens primarily comprised a platinum-based agent (cisplatin or nedaplatin) combined with either a taxane (paclitaxel) or a fluoropyrimidine (5-fluorouracil or capecitabine). Patients typically received 4 to 6 cycles of this combination therapy, followed by immune maintenance therapy until disease progression or unacceptable toxicity.

#### 2.2.2 Radiotherapy regimens

For radiotherapy, involved-field radiotherapy (IFRT) was strictly utilized for the primary esophageal tumor and clinically involved regional lymph nodes, deliberately avoiding elective nodal irradiation (ENI). This precise targeting strategy was adopted to minimize the irradiation volume, thereby sparing circulating lymphocytes and correlating with a reduced risk of severe radiation-associated lymphopenia to support the efficacy of concurrent immune checkpoint inhibitors.

In our cohort, 75 patients received RT to the primary tumor using conventionally fractionated intensity-modulated radiation therapy (IMRT), with a median delivered dose of 50.4 Gy (interquartile range [IQR]: 50.4–54.0 Gy; range: 50.4–60.0 Gy). Meanwhile, 67 patients received RT targeting specific oligometastatic lesions. Modalities such as IMRT or stereotactic body radiation therapy (SBRT) were applied based on the specific anatomical site and tumor volume, with an overall median dose of 50.0 Gy (IQR: 44.0–50.0 Gy; range: 30.0–60.0 Gy). The dose-fractionation schedules for metastatic sites were highly individualized to achieve optimal local control while respecting normal tissue constraints: lung metastases (e.g., 60 Gy in 15 fractions), bone metastases (e.g., 20–30 Gy in 5–10 fractions), brain metastases (e.g., 30–52.5 Gy in 10–15 fractions), liver metastases (e.g., 48 Gy in 12 fractions), adrenal metastases (e.g., 30 Gy in 10 fractions), and lymph node metastases (e.g., 50–60 Gy in 25–30 fractions).

#### 2.2.3 Definition of treatment sequencing

The primary intervention evaluated in this study was the temporal integration of local RT with systemic chemoimmunotherapy. Based on the exact clinical timing of RT initiation relative to the systemic therapy cycles, patients were categorized into three distinct strategies (1): RT-first group (n = 27): Radiotherapy was administered and completed prior to the initiation of chemoimmunotherapy, often necessitated by significant local symptoms requiring immediate palliative relief (2). Concurrent group (n = 87): Radiotherapy was seamlessly integrated within the first four cycles of induction chemoimmunotherapy (3). Sequential group (n = 28): Radiotherapy was administered as a consolidative local therapy only after the completion of at least four full cycles of chemoimmunotherapy.

### 2.3 Efficacy and toxicity assessment

Treatment efficacy was evaluated according to the Response Evaluation Criteria in Solid Tumors (RECIST) version 1.1. Responses were categorized as complete response (CR), partial response (PR), stable disease (SD), or progressive disease (PD). The disease control rate (DCR) was calculated as the proportion of patients who achieved CR, PR, or SD: DCR = ([CR + PR + SD]/total number of patients) × 100%.

Progression-free survival (PFS) was defined as the time from the initiation of the first treatment for oligometastatic disease (either radiotherapy or chemoimmunotherapy) to the date of disease progression or the last follow-up. Overall survival (OS) was defined as the time from the diagnosis of oligometastatic disease to the date of death from any cause or the last follow-up. Adverse events were graded using the Common Terminology Criteria for Adverse Events (CTCAE), version 5.0.

### 2.4 Statistical analysis

All statistical analyses were performed using R software (version 4.5.1). Baseline patient characteristics were described and stratified by treatment sequencing group (RT-first, Concurrent, and Sequential). Categorical variables are presented as frequencies and percentages, while continuous variables are presented as medians and interquartile ranges (IQR). The Standardized Mean Difference (SMD) was calculated to quantify the extent of imbalance in baseline covariates, with an SMD > 0.2 indicating a potential imbalance.

To robustly evaluate the association between treatment sequencing and survival while mitigating “confounding by indication,” we employed a causal inference framework utilizing Stabilized Inverse Probability of Treatment Weighting (IPTW). First, a Directed Acyclic Graph (DAG) was constructed based on existing clinical knowledge to formalize prior structural assumptions. By applying the back-door criterion, a minimal sufficient adjustment set was identified, comprising ECOG performance status, type of oligometastasis, number of metastatic lesions, and baseline neutrophil-to-lymphocyte ratio (NLR).

Rather than relying solely on traditional multivariable Cox regression—which is constrained by assumptions of linear additivity and is prone to overfitting when adjusting for numerous confounders relative to the event size—we utilized a dual-model propensity score strategy to create a balanced pseudo-population. Alongside a conventional multinomial logistic regression (LR) model, we developed a gradient boosting machine (GBM) model. As a non-parametric machine learning algorithm, GBM automatically captures complex non-linear relationships and multi-way interactions among the covariates without requiring manual specification, thereby achieving superior covariate balance. To prevent model overfitting and avoid the generation of extreme weights, the GBM tuning process was rigorously optimized using 5-fold cross-validation to determine the optimal number of decision trees.

Stabilized IPTW weights were then calculated. The model demonstrating the optimal covariate balance (defined as yielding the lowest SMDs among core confounders, targeting SMD < 0.2) was selected for all subsequent outcome analyses. This IPTW pipeline effectively separates the confounding adjustment (design phase) from the outcome evaluation (analysis phase).

The primary outcome analysis was conducted on the IPTW-weighted cohort. Weighted Kaplan-Meier curves were plotted to visualize survival distributions, and the weighted log-rank test was used to assess overall differences. To evaluate the independent clinical association of treatment timing with survival, a weighted multivariable Cox proportional hazards model was constructed to calculate adjusted hazard ratios (aHRs) and 95% confidence intervals (CIs). To adjust for potential residual confounding post-weighting, the specific radiotherapy site (primary vs. metastatic) was included as an additional covariate in this final model. Pairwise comparisons among the three groups were performed, and the Bonferroni correction was applied to control the overall Type I error rate, with a two-sided *P* < 0.0167 (0.05/3) considered statistically significant for these specific comparisons.

Finally, to explore independent prognostic factors, the treatment groups and other covariates were included in a multivariable Cox model built upon the IPTW-weighted cohort, with results visualized via a forest plot. Except for the Bonferroni-corrected pairwise comparisons, a two-sided *P* < 0.05 was considered statistically significant.

## 3 Results

### 3.1 Patient characteristics and balance after weighting

A total of 142 patients with oligometastatic esophageal squamous cell carcinoma were included in this study, comprising 27 (19.0%) in the RT-first group, 87 (61.3%) in the Concurrent group, and 28 (19.7%) in the Sequential group. The baseline demographic and clinical characteristics for all three groups are detailed in **Table 1**. The study cohort exhibited substantial data maturity at the time of analysis. Across the entire population (N = 142), a total of 94 deaths (66.2%) and 86 progression events (60.6%) were documented during the follow-up period. Specifically, the number of death events was 21 (77.8%) in the RT-first group, 52 (59.8%) in the Concurrent group, and 21 (75.0%) in the Sequential group. For progression-free survival, the number of events was 18 (66.7%), 51 (58.6%), and 17 (60.7%), respectively.

**Table 1:** Baseline characteristic table of patients before weighting.

Covariate	RT-first	Concurrent	Sequential	SMD
n	27	87	28	
Sex = Female (%)	5 (18.5)	24 (27.6)	9 (32.1)	0.211
Age_Group = >=60 (%)	22 (81.5)	68 (78.2)	16 (57.1)	0.364
ECOG = 1-2 (%)	24 (88.9)	81 (93.1)	21 (75.0)	0.342
Tumor_Location (%)				0.167
Upper	5 (18.5)	15 (17.2)	6 (21.4)	
Middle	14 (51.9)	51 (58.6)	13 (46.4)	
Lower	8 (29.6)	21 (24.1)	9 (32.1)	
Oligo_Type = Metachronous (%)	14 (51.9)	42 (48.3)	18 (64.3)	0.218
Met_Organ_Num = 2-3 (%)	4 (14.8)	13 (14.9)	6 (21.4)	0.115
Met_Lesion_Num = 4-5 (%)	4 (14.8)	10 (11.5)	4 (14.3)	0.066
RT_Site = Oligometastatic (%)	17 (63.0)	40 (46.0)	10 (35.7)	0.374
NLR (median [IQR])	2.83 [2.40, 3.42]	2.48 [1.96, 3.98]	2.32 [1.62, 3.63]	0.238

Prior to weighting, significant imbalances were present across the groups for several key covariates. Notably, the proportion of patients with an ECOG performance status of 1–2 was lower in the Sequential group (75.0%) compared to the Concurrent (93.1%) and RT-first groups (88.9%) (SMD = 0.342). Imbalances (all SMD > 0.2) were also observed for patient age, sex, type of oligometastasis, radiotherapy site, and baseline NLR. These initial imbalances highlight the potential for significant “confounding by indication” bias within this retrospective cohort.

**Figure 2:**
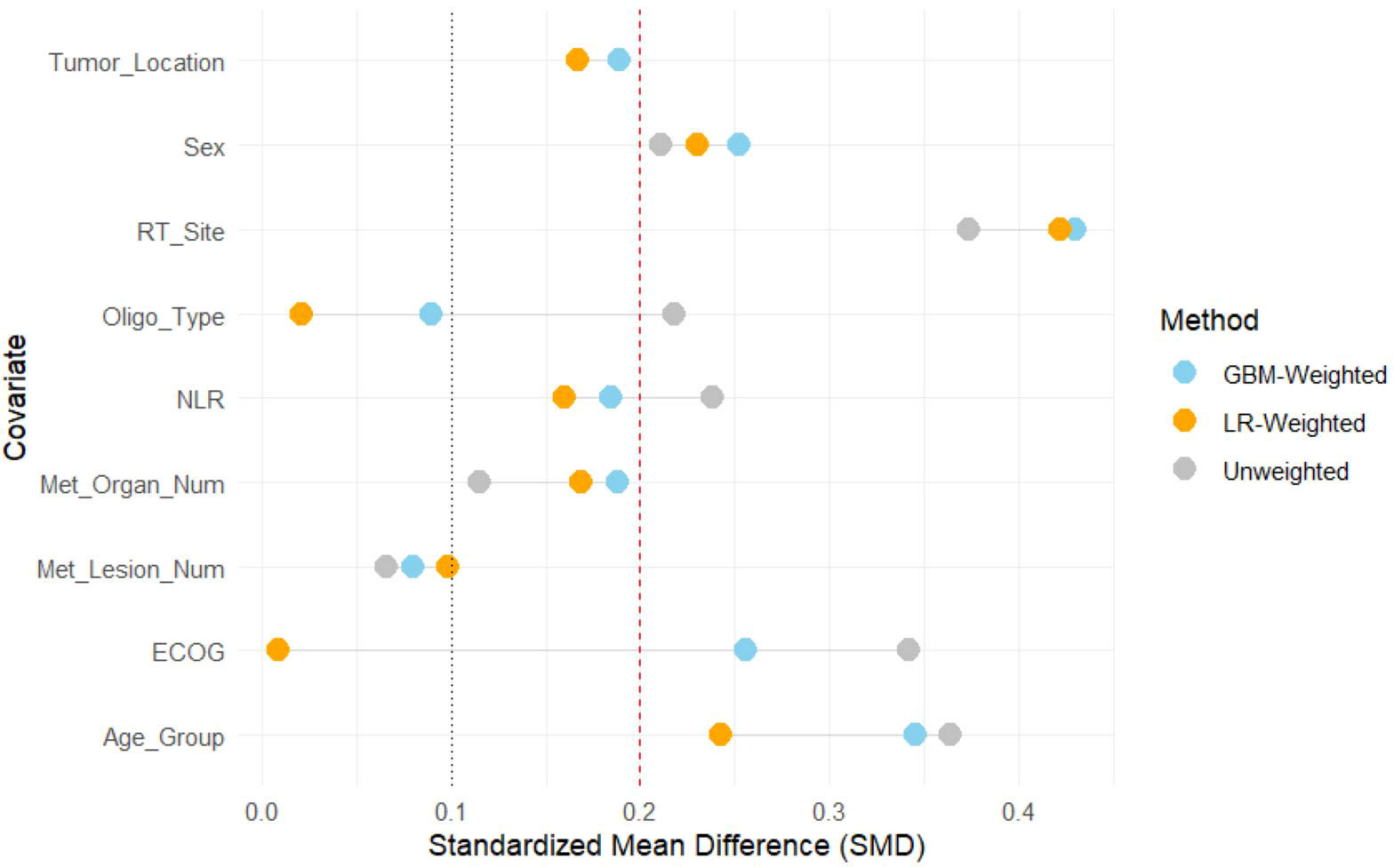
Balance of covariates before and after weighting comparison between GBM and logistic regression models. GBM-Weighted used Gradient Boosting Machine model; LR-Weighted used Multinomial Logistic Regression model.

To select the optimal propensity score model, we compared the covariate balancing performance of the multinomial logistic regression and GBM models. **Table 2** summarizes the SMD values before and after weighting, while **Figure 2** provides a visual comparison.

**Table 2:** Comparison of standardized mean differences for core covariates before and after weighting by two models.

Covariate	Unweighted SMD	SMD Post-Weighted by GBM	SMD Post-Weighted by LR
ECOG	0.342	0.256	0.009
RT_Site	0.374	0.430	0.422
Age_Group	0.364	0.346	0.243
NLR	0.238	0.185	0.160
Oligo_Type	0.218	0.090	0.021
Sex	0.211	0.252	0.230
Tumor_Location	0.167	0.189	0.167
Met_Organ_Num	0.115	0.188	0.169
Met_Lesion_Num	0.066	0.080	0.098

The results demonstrated that the logistic regression model achieved superior performance in balancing the key confounding factors. For instance, after weighting with the LR model, the SMD for ECOG performance status was reduced from 0.342 to a near-perfect 0.009, and the SMD for the type of oligometastasis decreased from 0.218 to 0.021. In contrast, the GBM model failed to adequately balance several covariates, including ECOG performance status (SMD = 0.256) and radiotherapy site (SMD = 0.430). Given that the traditional logistic regression achieved superior overall covariate balance and maintained model parsimony for our specific dataset, it was selected over the non-parametric GBM model to ensure the validity of the IPTW weights.

Therefore, all subsequent analyses were conducted using the stabilized IPTW calculated from the logistic regression model.

The follow-up for this study was conducted until August 2025, with a median follow-up time of 21.6 months (95% CI, 19.8–23.4 months). The disease control rate (DCR) was 66.7% in the RT-first group, 58.6% in the Concurrent group, and 60.7% in the Sequential group. There was no statistically significant difference in DCR among the three groups (χ² = 0.559, *P* = 0.756).

**Figure 3:**
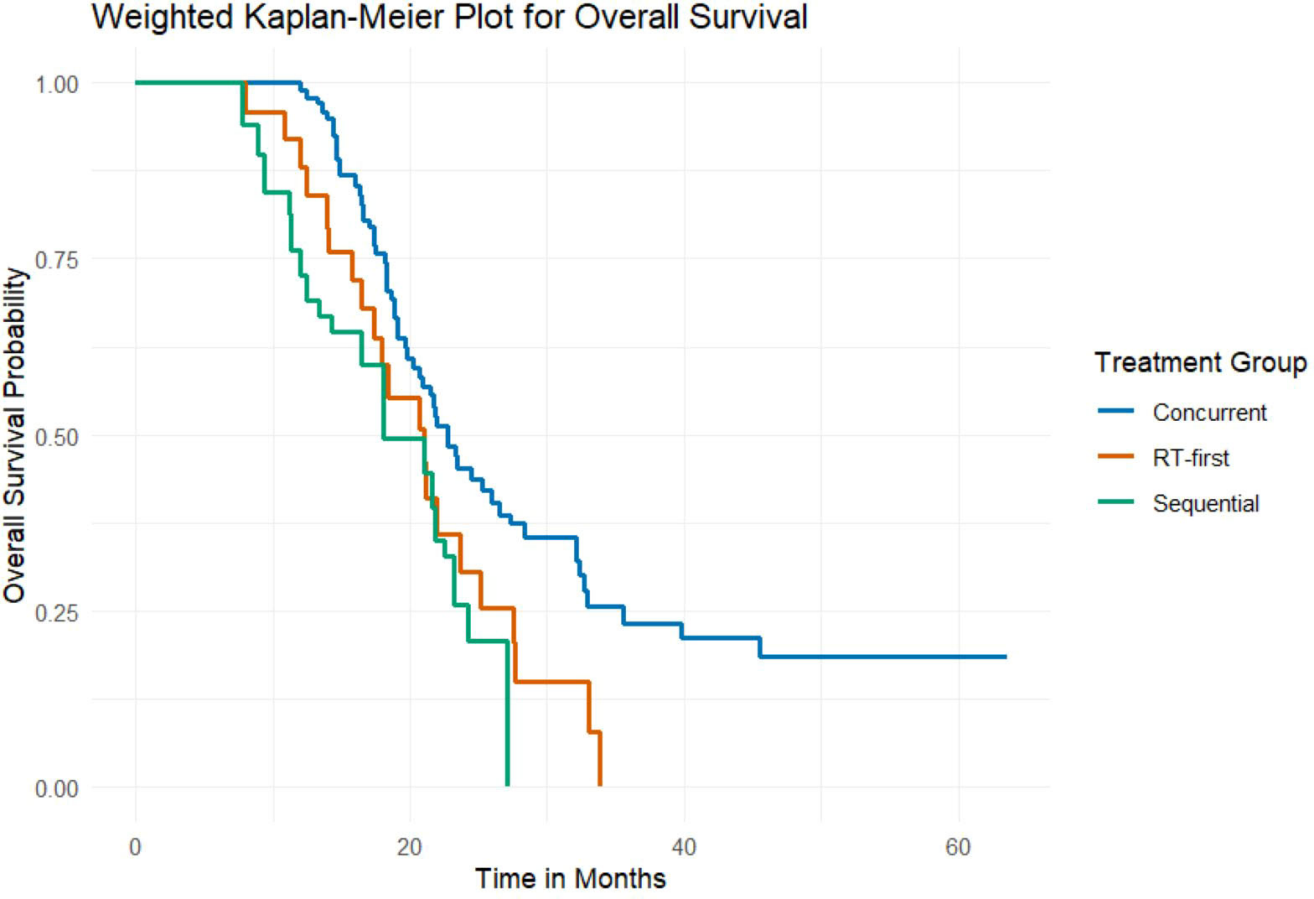
OS curves for three groups of patients.

The final weighted multivariate Cox regression analysis revealed a significant difference in overall survival (OS) among the three groups (weighted log-rank test, *P* = 0.018). The weighted Kaplan-Meier curves demonstrated superior OS in the Concurrent group (**Figure 3**). The median OS was 22.7 months (95% CI, 18.2–35.6) in the Concurrent group, compared to 21.0 months (95% CI, 15.8–27.5) in the RT-first group and 18.1 months (95% CI, 12.0–24.2) in the Sequential group.

**Figure 4:**
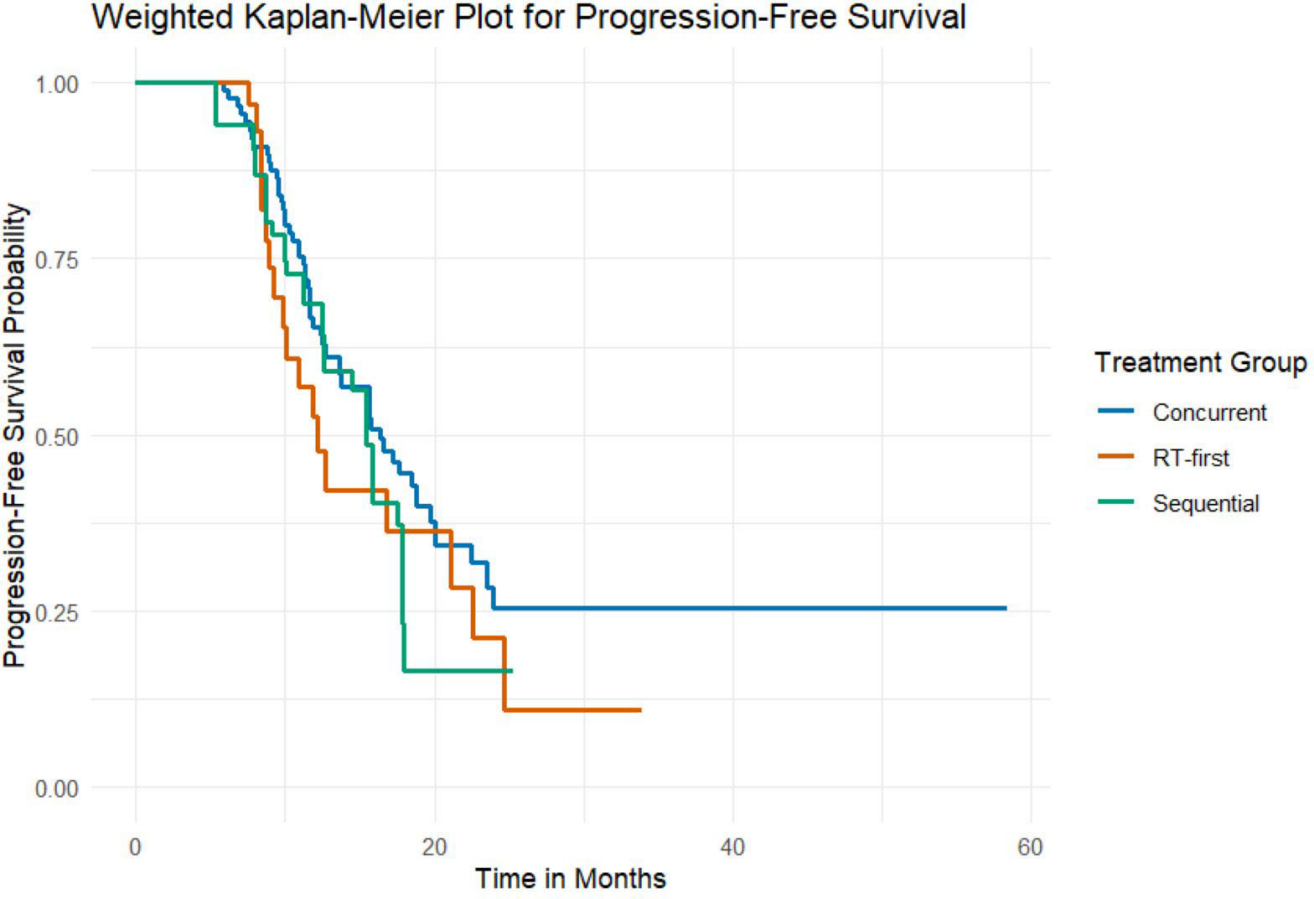
PFS curves for three groups of patients.

There was no significant difference in PFS among the three groups (weighted log-rank test, *P* = 0.314) (**Figure 4**). The median PFS was 16.4 months (95% CI, 11.2–not reached) for the Concurrent group, 12.2 months (95% CI, 8.9–22.6) for the RT-first group, and 15.4 months (95% CI, 10.0–17.8) for the Sequential group.

A significant overall difference in OS was observed among the treatment groups (global Wald test, *P* = 0.02). Pairwise comparisons revealed that the Concurrent group had a significantly lower risk of death compared to the Sequential group (aHR = 0.47; 95% CI, 0.27–0.83; *P’* = 0.010), a difference that remained significant after Bonferroni correction (P’ < 0.0167). Although a strong trend toward improved survival was also observed for the Concurrent group compared to the RT-first group (aHR = 0.58; 95% CI, 0.37–0.92; *P’* = 0.022), this difference did not meet the significance threshold after Bonferroni correction (**Table 3**).

**Table 3:** Weighted multivariable Cox regression analysis of the impact of treatment sequencing on OS and PFS.

Characteristic	aHR	95% CI	P-value
OS			
Treatment Group			
Concurrent vs. RT-first (reference)	0.58	0.37 - 0.92	0.022
Sequential vs. RT-first (reference)	1.22	0.65 - 2.29	0.526
Sequential vs. Concurrent (reference)	2.11	1.20 - 3.70	0.010
RT Site			
Oligometastasis vs. Primary lesion (reference)	1.07	0.71 - 1.63	0.734
PFS			
Treatment Group			
Concurrent vs. RT-first (reference)	0.70	0.40 - 1.23	0.216
Sequential vs. RT-first (reference)	0.96	0.47 - 1.94	0.899
RT Site			
Oligometastasis vs. Primary lesion (reference)	0.97	0.63 - 1.51	0.903

To identify the independent prognostic factors for overall survival, we first conducted a univariate weighted Cox regression analysis on 10 potential prognostic variables. Based on a pre-specified screening criterion of P < 0.20, a total of six variables were selected as candidates for the multivariate model: treatment group, sex, age group, ECOG performance status, tumor location, and baseline NLR (**Table 4**).

**Table 4:** Univariable and multivariable weighted Cox regression analysis for OS.

Characteristic	Univariable analysis		Multivariable analysis	
	P	HR (95%CI)	P	aHR (95%CI)
Treatment Group	0.009		<0.001	
RT-first		Reference		Reference
Concurrent	0.021	0.58 (0.36 - 0.92)	0.007	0.53 (0.33 - 0.84)
Sequential	0.546	1.21 (0.65 - 2.28)	0.447	1.29 (0.67 - 2.50)
Age	0.027	0.59 (0.37 - 0.94)	0.028	0.59 (0.37 - 0.94)
NLR	0.061	1.01 (1.00 - 1.03)	0.002	1.02 (1.01 - 1.03)
Tumor Location	0.300		0.030	
Upper		Reference		Reference
Middle	0.138	1.51 (0.88 - 2.61)	0.037	1.93 (1.04 - 3.58)
Lower	0.195	1.54 (0.80 - 2.98)	0.074	1.96 (0.94 - 4.11)
Sex	0.088	1.46 (0.95 - 2.24)	–	–
ECOG	0.049	0.57 (0.33 - 0.99)	–	–

Variables for the multivariate model were selected via backward elimination from candidate variables with a p-value < 0.20 in the univariate analysis. The final multivariate model included treatment group, age group, NLR, and tumor location. A dash (—) indicates that the variable was not included in the final multivariate model.

Subsequently, these six candidate variables were included in a multivariate weighted Cox model and subjected to a backward stepwise selection process with a removal criterion of P > 0.10. During this process, ECOG performance status (*P* = 0.401 in the full model) and sex (*P* = 0.252 in a subsequent model) were sequentially eliminated from the model.

**Figure 5:**
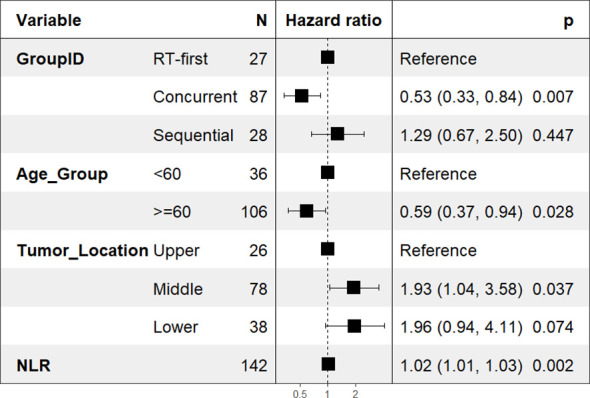
Forest plot of the final multivariable Cox regression model for independent prognostic factors of OS.

In the final weighted multivariate Cox regression analysis for prognosis, four variables were identified as independent predictors of overall survival (**Figure 5**).

Concurrent treatment timing remained a strong protective factor (vs. the RT-first group; aHR = 0.53, 95% CI, 0.33–0.84; P = 0.007). Additionally, a higher baseline NLR (aHR per unit increase = 1.02; 95% CI, 1.01–1.03; P = 0.002) and a tumor location in the middle thoracic esophagus (vs. upper thoracic; aHR = 1.93; 95% CI, 1.04–3.58; P = 0.037) were independent risk factors for mortality. Interestingly, an age of ≥60 years was also found to be an independent protective factor (aHR = 0.59; 95% CI, 0.37–0.94; P = 0.028).

### 3.2 Safety analysis

The treatment regimens were well-tolerated by all patients in the study cohort. No patients discontinued therapy due to intolerable adverse events. All observed treatment-related adverse events (TRAEs) were mild to moderate in severity (predominantly Grade 1–2) and were manageable with timely intervention.

The most common toxicities included myelosuppression, pneumonitis, and abnormal liver or renal function. There was no statistically significant difference in the overall incidence of toxicities among the three treatment groups (P > 0.05) (**Table 5**).

**Table 5:** Incidence of adverse reactions in the three groups.

Group	Myelosuppression	Radiation Esophagitis	Hepatic and renal dysfunctionn	Pneumonia	Thyroid dysfunction
RT-first (n=27)	9 (33.33)	19 (70.37)	3 (11.11)	2 ( 7.41)	2 (7.41)
Concurrent (n=87)	28 (32.18)	57 (65.52)	5 (5.75)	3 (3.45)	1 (1.15)
Sequential (n=28)	8 (28.57)	20 (71.43)	5 (17.86)	1 (3.57)	2 (7.14)
X^2^	0.116	0.091	4.098	0.800	3.588
P	0.943	0.955	0.129	0.669	0.166

### 3.3 Sensitivity analyses and patterns of failure

**Figure 6:**
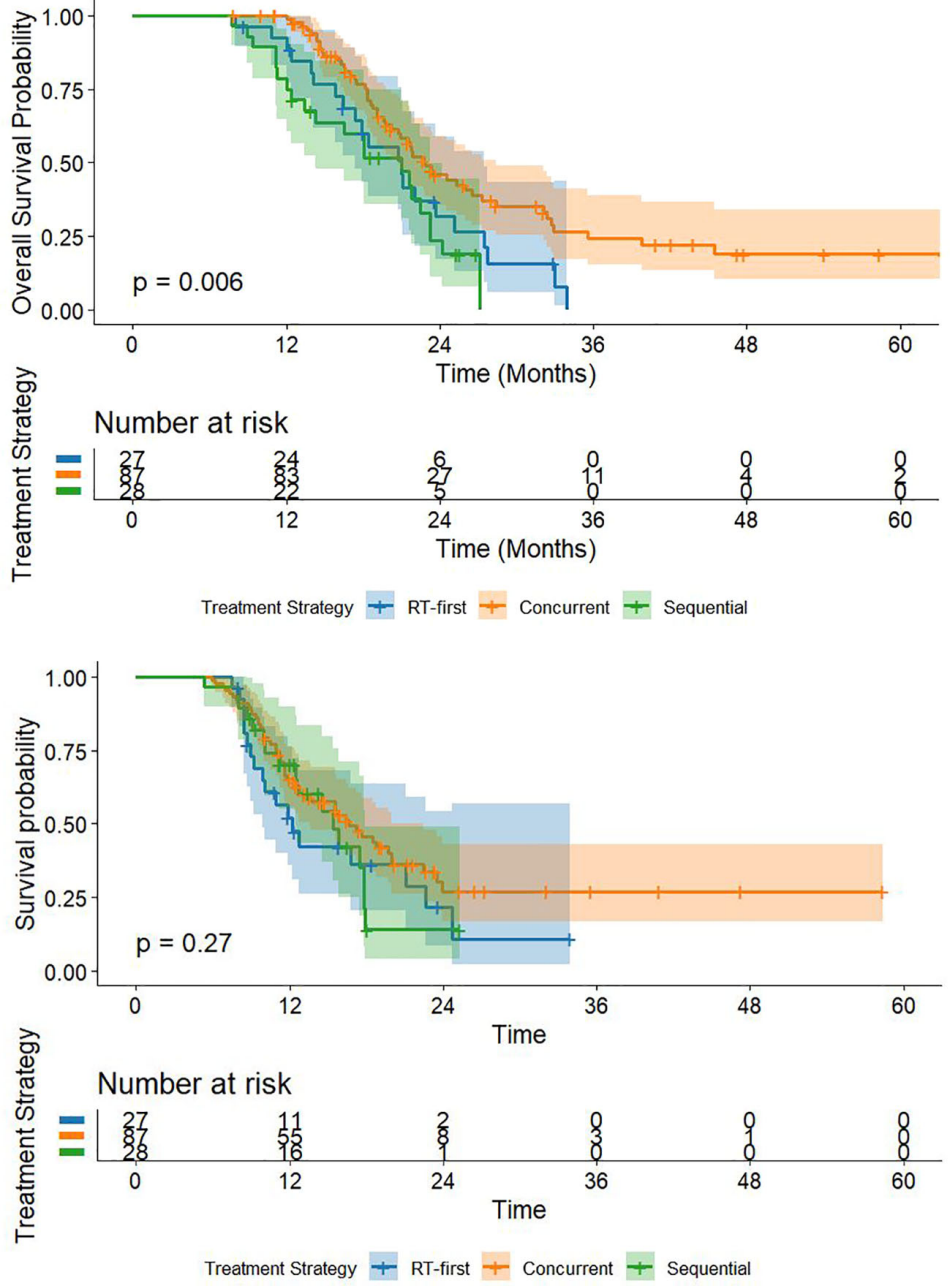
The survival curves of OS and PFS before weighting of the three group.

To transparently present the real-world temporal distribution of RT initiation, we plotted the exact number of patients starting RT at each specific CIT cycle (**Supplementary Figure 1**). To further address potential temporal heterogeneity within the 4-cycle window of the Concurrent group, we conducted a sensitivity analysis stratifying these 87 patients into ‘Early-Concurrent’ (RT during cycles 1-2, n = 46) and ‘Late-Concurrent’ (RT during cycles 3-4, n = 41). The Kaplan-Meier analysis (**Supplementary Figure 1**) revealed no significant difference in OS between the two sub-cohorts (median OS: 21.5 vs. 23.4 months, Log-rank P = 0.808), indicating that RT integration anywhere within the first 4 cycles yields comparable survival outcomes (An analysis of the unweighted raw data also shows the same trend as the weighted data, **Figure 6**.).

Furthermore, to investigate the underlying clinical reasons for the observed discordance between the significant OS benefit and non-significant PFS, we analyzed the patterns of first failure and subsequent salvage treatments. The distribution of local progression, distant oligometastatic, and polymetastatic dissemination showed no statistically significant differences across the RT-first, Concurrent, and Sequential groups (x^2^ = 4.319, *P* = 0.374) (**Supplementary Table 1**). Additionally, upon documented disease progression, the proportions of patients who successfully transitioned to standard second-line salvage therapies (e.g., single-agent chemotherapy or targeted therapy) were highly comparable across the three cohorts: 59.3% (16/27) in the RT-first group, 58.6% (51/87) in the Concurrent group, and 60.7% (17/28) in the Sequential group.

Finally, to account for the potential distinct immunomodulatory effects of various immune checkpoint inhibitors, we performed a sensitivity analysis by including the specific PD-1 inhibitor agent (camrelizumab, tislelizumab, or sintilimab) as a covariate in the Cox regression model. The specific type of immunotherapy agent was not significantly associated with overall survival (P > 0.05) and did not alter the independent prognostic significance of the RT timing strategy (**Supplementary Table 2**).

## 4 Discussion

While current clinical practice guidelines provide therapeutic recommendations for each stage of esophageal cancer, the distinct stage of oligometastatic disease is often overlooked. The oligometastatic state is considered an intermediate phase between localized primary disease and widespread metastases and may be characterized by a more indolent biological behavior. Correspondingly, patients with oligometastatic esophageal cancer have a better prognosis than those with extensive metastatic disease, and evidence suggests that the addition of local radiotherapy to standard chemoimmunotherapy can improve patient outcomes (19).

In line with these evolving paradigms, a growing body of literature has extensively explored the combination of RT with immunotherapy or chemoimmunotherapy across various advanced malignancies. Beyond oligometastatic models, the addition of RT to immune checkpoint inhibitors has demonstrated substantial survival benefits in advanced non-small cell lung cancer (e.g., the PEMBRO-RT trial) and melanoma, primarily by overcoming primary resistance to ICIs and augmenting systemic tumor control (20). More importantly, in the specific context of stage IV esophageal cancer, several recent retrospective studies have confirmed the clinical efficacy of combining local radiotherapy with systemic immunotherapy or chemoimmunotherapy. For instance, recent real-world cohorts of patients with metastatic ESCC receiving first-line PD-1 inhibitors plus chemotherapy have shown that the addition of local RT to the primary tumor or metastatic lesions significantly prolongs both PFS and OS compared to systemic chemoimmunotherapy alone, with manageable toxicity profiles (21) (18). These studies collectively underscore that the triple combination (RT + Chemo + ICI) is highly active and synergistic in advanced ESCC. However, while these previous retrospective studies robustly validated the efficacy of the combination itself, they largely treated RT as a monolithic intervention without rigorously stratifying the timing of its administration. This leaves a critical void in clinical practice regarding exactly when to introduce RT during the chemoimmunotherapy course, which our study directly addresses.

To further contextualize our findings, we compared our survival outcomes with those reported in recent retrospective studies evaluating radiotherapy combined with immunotherapy-based regimens. In the present study, the median overall survival (mOS) of the Concurrent RT group reached 22.7 months (95% CI, 18.2–35.6), which appears numerically superior to outcomes reported in prior real-world cohorts. For example, Wu et al. (2023) reported a median OS of 11.9 months (95% CI, 8.61–19.2) in patients receiving immunotherapy with or without radiotherapy in metastatic ESCC. Similarly, Amin et al. (2024) demonstrated that chemoradiation combined with immunotherapy achieved a median OS of 12.4 months (95% CI, 11.7–13.3), significantly longer than chemoradiation alone but still shorter than the survival observed in our Concurrent strategy. Similarly, Tasoudis et al. (2024) demonstrated the feasibility of integrating immunotherapy into multimodal chemoradiation strategies, although their study primarily focused on neoadjuvant settings rather than metastatic disease. This difference may be partly attributed to the oligometastatic disease setting in our cohort, which is biologically distinct from widely metastatic populations included in previous studies.

Although cross-study comparisons should be interpreted cautiously due to differences in patient selection, disease burden, and treatment protocols, these findings collectively support the benefit of integrating radiotherapy into immunotherapy-based treatment. Importantly, unlike these previous studies, which primarily evaluated the addition of radiotherapy itself, our study uniquely focused on the temporal sequencing of radiotherapy. By directly comparing RT-first, concurrent RT, and sequential RT strategies within the same cohort, we demonstrated that early concurrent radiotherapy was associated with the most favorable survival outcomes. These findings extend previous retrospective evidence by providing clinically actionable insight into the optimal timing of radiotherapy initiation.

Specific to oligometastatic esophageal cancer, the ESO-Shanghai 13 trial—a phase II, randomized, multicenter clinical trial involving 104 patients—provided strong evidence for this approach. The trial reported that the median PFS for patients receiving both systemic and local therapy was 15.3 months, significantly longer than the 6.4 months observed in the systemic-therapy-alone arm (HR, 0.26; P < 0.0001), without an increased incidence of Grade ≥3 treatment-related adverse events (10). Notably, the survival benefit observed in our Concurrent group appears comparable to the improved progression-free survival reported in the ESO-Shanghai 13 trial, further reinforcing the therapeutic value of integrating local therapy into systemic treatment strategies. The forthcoming ESO-Shanghai 20 trial (NCT05506248) is set to directly evaluate the survival benefit of adding local therapy in the modern context of chemoimmunotherapy (22). Should this trial yield positive results, the combination of radiotherapy with chemoimmunotherapy could become the recommended first-line standard of care for oligometastatic ESCC.

The mechanisms underlying the synergistic efficacy of this combination therapy have been elucidated and can be summarized as follows (1): Immunogenic Cell Death (ICD) and Antigen Release: Both chemotherapy and radiotherapy can induce ICD in tumor cells, leading to the release of tumor-associated antigens (TAAs) and damage-associated molecular patterns (DAMPs). This process activates dendritic cells (DCs), enhances their antigen-presenting capabilities, and effectively creates an “*in situ* vaccine” that generates targets for immunotherapy (23) (2). Tumor Microenvironment (TME) Remodeling: Radiotherapy can favorably remodel the TME, for instance, by inducing hypoxia, which can enhance the effects of immunotherapy. Conversely, immune checkpoint inhibitors (ICIs) not only unleash T cells to attack tumor cells but can also modulate the TME by normalizing tumor vasculature, thereby increasing radiosensitivity (24) (3). The Abscopal Effect and Systemic Immune Activation: By triggering the release of TAAs and pro-inflammatory cytokines, radiotherapy can activate a systemic immune response. The addition of chemoimmunotherapy can potentiate this systemic anti-tumor response, often referred to as the abscopal effect (25). However, despite this understanding of the underlying mechanisms, the optimal timing for integrating local radiotherapy with a chemoimmunotherapy regimen remains to be definitively established.

This study provides a preliminary exploration into the optimal timing of local radiotherapy, and our results indicate that concurrent administration with chemoimmunotherapy prolongs OS. The potential mechanisms for this finding are as follows: the antigen release, inflammatory storm, and immune cell recruitment induced by chemoradiation create a relatively transient “window of opportunity.” Administering ICIs concurrently within this window may most effectively capitalize on these immunostimulatory signals, unleashing nascent T-cell responses from an inhibited state to achieve a maximal synergistic killing effect.

Furthermore, the three modalities may engage in a positive feedback loop: chemotherapy can deplete immunosuppressive cells, immunotherapy can reverse T-cell exhaustion, and radiotherapy releases antigens while activating inflammatory signals. This combined approach can also promote the normalization of tumor vasculature, thereby improving T-cell infiltration and sustaining long-term immunological memory, which ultimately translates into a prolonged survival benefit.

Interestingly, while our study demonstrated a significant OS benefit for the Concurrent group, the PFS difference was not statistically significant. This discordance required careful interpretation. Our extensive analysis of post-progression data revealed that neither the anatomical patterns of first failure (P = 0.374) nor the proportions of patients receiving subsequent second-line salvage therapies (59-61% across all arms) differed significantly. This balanced post-progression landscape essentially rules out the confounding effect of unequal subsequent care.

Therefore, we hypothesize that the profound OS benefit of the Concurrent strategy is rooted in the superior quality and durability of the immune response generated during first-line intervention. Furthermore, the significant survival advantage observed exclusively in the Concurrent group, but not in the RT-first or Sequential groups, highlights the critical spatiotemporal dynamics of the tumor-immune cycle. We hypothesize that this clinical divergence is driven by three distinct immunological scenarios:

First, regarding the RT-first strategy (Immune Depletion), upfront radiotherapy targeting macroscopic lesions inevitably exposes a significant volume of circulating blood to radiation, frequently resulting in severe radiation-induced lymphopenia (RIL). This inadvertently ablates the host’s naive and effector T-cell pools. Because the immunogenic cell death (ICD) and antigen release induced by RT are transient, initiating PD-1 blockade only after the completion of RT means the optimal ‘priming window’ has closed, and the depleted immune system is unable to mount a robust systemic response (13).

Second, regarding the Sequential strategy (Immune Exhaustion), delaying local RT until after extensive cycles of systemic therapy allows the tumor to continuously edit its microenvironment. Prolonged exposure to macroscopic tumor burden and prior systemic agents can drive the infiltrating lymphocytes into a state of profound T-cell exhaustion and acquired resistance. Consequently, when late-course RT is finally administered, the exhausted immune microenvironment is refractory to the newly released antigens, blunting any potential abscopal effect (26).

In contrast, the Concurrent strategy (Spatiotemporal Synergy) aligns precisely with the optimal therapeutic window. By integrating RT during the early cycles of chemoimmunotherapy, the massive release of tumor neoantigens and pro-inflammatory cytokines perfectly overlaps with the peak pharmacological activity of the immune checkpoint inhibitors. This immediate PD-1 blockade prevents early T-cell apoptosis, rescues them from exhaustion exactly at the moment of antigen presentation, and drives a robust clonal expansion. This synergistic interplay ultimately translates into durable immunological memory and the superior overall survival observed in our study (27).

The association between the NLR and cancer prognosis is well-documented in previous research, consistent with the findings of our study. For instance, in a study of patients with advanced non-small cell lung cancer treated with immune checkpoint inhibitors, a high post-treatment NLR (P = 0.004) was an independent prognostic factor for shorter OS (28). Similarly, a meta-analysis in melanoma demonstrated that a high NLR is likely associated with poorer patient outcomes (29).

The biological basis for this association likely relates to the distinct roles of neutrophils and lymphocytes within the tumor microenvironment. Neutrophils can facilitate tumor progression through various mechanisms, including promoting cell migration (30), inducing immunosuppression (31), and stimulating angiogenesis (32). Supporting their role as a potential biomarker, Fridlender et al. (33) found that blocking the TGF-β receptor recruited neutrophils to the TME and promoted tumor progression. In contrast, lymphocytes, such as the γδ T cells that Cui et al. (34) confirmed to have anti-tumor activity in esophageal cancer, are crucial for anti-cancer immunity. This immune response can be compromised by factors such as radiotherapy, which has been shown to inhibit CD8+ T cell infiltration and activate immunosuppression (35), and immunosenescence associated with aging (36). Furthermore, radiotherapy-induced lymphopenia is a known factor associated with adverse prognoses (37). Taken together, these multifaceted factors likely explain why a high pretreatment NLR is indicative of a poorer OS in this patient population.

An unexpected yet statistically robust finding of this study was that an age of ≥60 years was identified as an independent protective factor for overall survival. The most probable explanation for this is selection bias and unmeasured confounding. Older patients eligible for and able to tolerate high-intensity combination therapy may represent a distinct subpopulation with superior functional status and physiologic reserve not fully captured by traditional metrics like the ECOG score (38). Conversely, younger patients may present with more aggressive tumor biology, leading to earlier disease onset and a poorer prognosis (39).

Furthermore, there may be inherent differences in tumor biology and the immune microenvironment across age groups (40). For instance, tumors in older patients might possess more indolent molecular features. Alternatively, the “inflammaging” immune background in the elderly could, paradoxically, enhance the response to this specific combination therapy. Therefore, the underlying cause of this finding is likely multifactorial and requires further validation and elucidation in future prospective studies that include the analysis of biological samples.

In the present study, the most common treatment-related adverse events (TRAEs) were predominantly Grade 1–2 and included myelosuppression, hepatic and renal dysfunction, radiation esophagitis, radiation pneumonitis, and thyroid dysfunction. There was no statistically significant difference in the incidence of toxicities among the three groups (*P* > 0.05).

These safety findings are consistent with prior research, which has also shown no significant difference in the incidence of TRAEs between chemoimmunotherapy combined with local radiotherapy and chemoimmunotherapy alone. For instance, in a retrospective study of patients with brain metastases from non-small cell lung cancer after first-line treatment, the incidence of Grade ≥3 AEs was numerically higher in the combination therapy group compared to the monotherapy group (12% vs. 4%), but this difference was not statistically significant (*P* = 0.269) (41). Furthermore, both the ESO-Shanghai 13 trial (10) and a Phase II trial of chemoradio-immunotherapy for oligometastatic ESCC after first-line treatment failure (33) concluded that combining radiotherapy with chemoimmunotherapy is safe and feasible, which aligns with the conclusions of our study.

Taken together, while previous retrospective studies have established the benefit of combining radiotherapy with systemic therapy, our study uniquely advances the field by clarifying the optimal temporal integration of radiotherapy, thereby addressing a clinically relevant question that has remained unresolved in prior literature.

## 5 Limitations

Several limitations of this study must be acknowledged. First, the relatively small sample size, particularly in the sequential and RT-first cohorts, may limit the statistical power and generalizability of our findings. Second, there was inherent heterogeneity in the radiotherapy modalities (IMRT vs. SBRT) and dose-fractionation regimens across different target sites (primary vs. varying metastatic organs). Biologically, it is well-documented that different fractionation schemes exert distinct immunomodulatory effects; for instance, ablative doses (SBRT) may trigger a more robust release of tumor neoantigens compared to conventional fractionation. Although we adjusted for the radiotherapy site (primary vs. metastatic) in our IPTW and multivariable Cox models to mitigate baseline confounding, sample size limitations precluded robust sub-group analyses stratified by specific RT doses or the exact metastatic organ irradiated. Future prospective trials with standardized RT protocols and rigorous translational research are imperative to determine the optimal dose-fractionation for synergizing with chemoimmunotherapy in ESCC.

Finally, while we employed a robust causal inference framework utilizing DAGs and IPTW to mitigate baseline imbalances, the validity of estimating causal effects from observational data fundamentally relies on the assumption of no unmeasured confounding. Since this assumption is untestable, our findings must be conservatively interpreted as strong clinical associations rather than definitive causality.

## 6 Conclusions

In conclusion, this multicenter retrospective study demonstrates that among patients with oligometastatic esophageal squamous cell carcinoma receiving first-line chemoimmunotherapy, the concurrent initiation of local radiotherapy (within the first four cycles) is significantly associated with prolonged overall survival compared to RT-first or sequential approaches. The concurrent strategy appears to offer an optimal temporal window that maximizes clinical benefit without significantly increasing severe toxicities. However, given the inherent limitations of a retrospective design, these findings reflect strong clinical associations rather than definitive causality. Future prospective, randomized controlled trials are urgently warranted to validate these associations and definitively establish the optimal sequencing paradigm in clinical practice.

## Data Availability

The raw data supporting the conclusions of this article will be made available by the authors, without undue reservation.
