# Supplementary material for: Preliminary exploration of the optimal timing of chemoimmunotherapy combined with radiotherapy for oligometastatic esophageal squamous cell carcinoma and analysis of prognostic factors: a multicenter retrospective study
Source: Front Oncol. 2026 May 18;16:1734354. doi: 10.3389/fonc.2026.1734354 (PMC13222794; doi:10.3389/fonc.2026.1734354)
Supplement: Supplementary file 1 [file DataSheet1.doc]

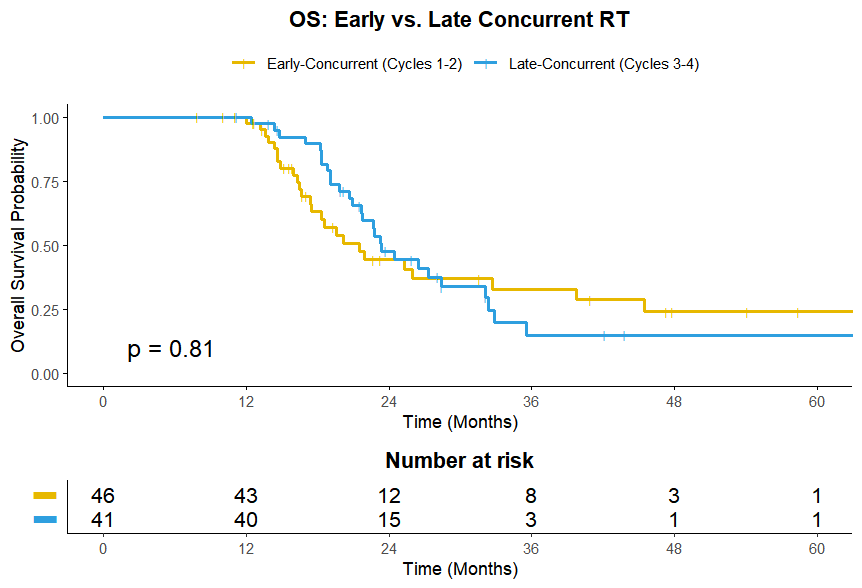


Supplementary Figure S1. Sensitivity analysis of OS between the early-concurrent (RT initiated within 1-2 cycles of CIT) and late-concurrent (RT initiated within 3-4 cycles of CIT) subgroups.


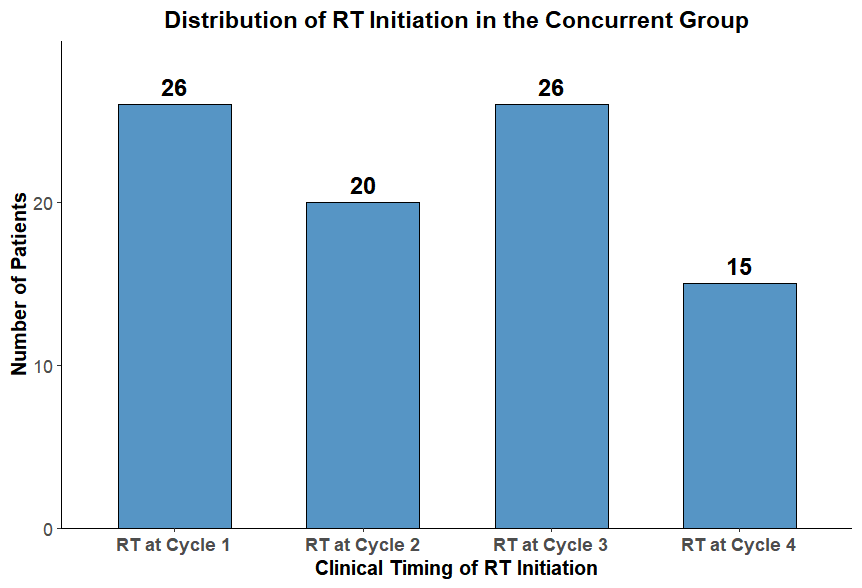


Supplementary Figure S2. Distribution of radiotherapy initiation timing relative to chemoimmunotherapy cycles in the Concurrent group.

Supplementary Table S1. Patterns of first failure and subsequent second-line treatments across the three treatment groups.

|  | Local Distant | Oligometastatic | Polymetastatic | Second-line treatment |
| --- | --- | --- | --- | --- |
| RT-first（n=27） | 7 | 8 | 1 | 16（59.26） |
| Concurrent（n=87） | 15 | 24 | 12 | 51（58.62） |
| Sequential（n=28） | 3 | 9 | 5 | 17（60.71） |

Supplementary Table S2. Sensitivity analysis of OS incorporating specific immune checkpoint inhibitors as covariates in the multivariable Cox regression model.

| Variables | HR | 95% CI | P |
| --- | --- | --- | --- |
| Treatment Group |  |  |  |
| RT-first | Reference | - | - |
| Concurrent | 0.595 | 0.354 – 0.999 | 0.049 |
| Sequential | 1.258 | 0.682 – 2.319 | 0.461 |
| Immunotherapy |  |  |  |
| Camrelizumab | Reference | - | - |
| Tislelizumab | 1.016 | 0.637 – 1.618 | 0.946 |
| Sintilimab | 1.029 | 0.315 – 3.352 | 0.962 |

Note: The model evaluates whether the specific type of PD-1 inhibitor confounds the survival benefit of the RT timing strategy. The RT timing strategy (Concurrent group) remains an independent prognostic factor even after adjusting for the specific immunotherapy agents.
